# Indoor 2D Positioning Method for Mobile Robots Based on the Fusion of RSSI and Magnetometer Fingerprints

**DOI:** 10.3390/s23041855

**Published:** 2023-02-07

**Authors:** Peter Sarcevic, Dominik Csik, Akos Odry

**Affiliations:** 1 Department of Mechatronics and Automation, Faculty of Engineering, University of Szeged, Moszkvai krt. 9, 6725 Szeged, Hungary; 2 Doctoral School of Applied Informatics and Applied Mathematics, Óbuda University, Bécsi str. 96/b, 1034 Budapest, Hungary

**Keywords:** position estimation, indoor positioning, localization, mobile robots, sensor fusion, RSSI, magnetometer, fingerprint

## Abstract

Received signal strength indicator (RSSI)-based fingerprinting is a widely used technique for indoor localization, but these methods suffer from high error rates due to various reflections, interferences, and noises. The use of disturbances in the magnetic field in indoor localization methods has gained increasing attention in recent years, since this technology provides stable measurements with low random fluctuations. In this paper, a novel fingerprinting-based indoor 2D positioning method, which utilizes the fusion of RSSI and magnetometer measurements, is proposed for mobile robots. The method applies multilayer perceptron (MLP) feedforward neural networks to determine the 2D position, based on both the magnetometer data and the RSSI values measured between the mobile unit and anchor nodes. The magnetic field strength is measured on the mobile node, and it provides information about the disturbance levels in the given position. The proposed method is validated using data collected in two realistic indoor scenarios with multiple static objects. The magnetic field measurements are examined in three different combinations, i.e., the measurements of the three sensor axes are tested together, the magnetic field magnitude is used alone, and the Z-axis-based measurements are used together with the magnitude in the X-Y plane. The obtained results show that significant improvement can be achieved by fusing the two data types in scenarios where the magnetic field has high variance. The achieved results show that the improvement can be above 35% compared to results obtained by utilizing only RSSI or magnetic sensor data.

## 1. Introduction

Indoor positioning techniques are utilized in a large variety of applications, such as emergency management [1], smart energy management [2], heating, ventilation, and air conditioning (HVAC) control systems [3], occupancy detection [4], and industrial monitoring [5]. They can be used to determine the position of humans, mobile robots, and objects, etc. In mobile robot applications, the localization problem is the first critical task which needs to be solved in control algorithms, since it directly influences their success. Localization provides the robot pose estimate using a sensor fusion framework, where generally relative and absolute poses are fused with probabilistic approaches. The accurate estimation of the absolute position plays a crucial role in these sensor fusion-based localization methods. In an indoor environment, the GPS cannot provide reliable measurement data, so other technologies have to be considered. Various technologies can be used for this task, such as cameras, LiDAR [6], and radio communication modules [7], etc. The fusion of these technologies was also widely used in related research [8].

In wireless sensor networks (WSN), wireless signal-based techniques are widely used in indoor positioning [9]. These techniques can be based on different types of extracted parameters, such as time of arrival (ToA), time of flight (ToF), angle of arrival (AoA), time difference of flight (TDoF), time difference of arrival (TDoA), received signal strength indicator (RSSI), and channel state information (CSI) [10]. The used methods can be divided into two major groups: geometric and fingerprint-based methods [10]. Geometric approaches include trilateration, multilateration, and triangulation methods. Various measurement parameters can be used in the case of these methods, such as ToA, ToF, or AoA.

The most often applied data type is the RSSI, which can be read from the transceiver modules. In the case of both method families, the position is estimated based on RSSI measurements between multiple anchor nodes with a known position and a unit with an unknown position. In geometric-based algorithms, the RSSI value is converted to distance based on an appropriate model, and then the position is estimated using trilateration [11]. In the case of fingerprint-based methods, measurements are collected in a room at fixed points with a given resolution, then a pattern recognition or pattern-matching algorithm (e.g., artificial neural networks (ANN), k-nearest neighbor (k-NN), etc. [12]) is trained using the measurement data, which is later utilized to determine the position of the unknown point [10,13,14]. Deep learning-based methods also offer an alternative solution to the problem [15,16], but they require much more computational and memory capacity than other methods. This can affect the real-time operation of the system, which is particularly important in cases of their use on mobile robots, where the embedded system must perform other tasks also. RSSI-based technologies are affected by various reflections, interferences and noises, which can influence their localization performance significantly, especially in indoor applications [17]. Some methods even utilize the trilateration and fingerprint-based results together in the fusion algorithm to improve the performance [18], while other methods fuse fingerprinting and time-based techniques [19].

In an outdoor environment, magnetic sensors are mainly utilized as compasses, but indoors they are almost useless in such form, since there are several hard iron and soft iron sources that affect the measurements [20]. This geomagnetism-based technique has attracted considerable attention in recent years [21,22], and has been shown to be a promising technology for indoor localization, since the field is more stable with much lower random fluctuation as compared to other signals [23,24]. Each building has its own unique ambient magnetic field, and if these local anomalies have sufficient variability, then they can be utilized during indoor localization [25].

Various approaches were previously proposed for indoor localization using geomagnetism, but these mainly consider human localization with smartphone-based systems [21,26]. The proposed methods are usually based on a sequence of geomagnetic data, which are generally fused with other data, such as WiFi [23,27,28,29,30], CSI [31], inertial measurement units (IMU) [32], pedestrian dead reckoning (PDR) [28,30,33], or pedometers [34]. In [33], a fusion algorithm was proposed which combined PDR and matching in a magnetic fingerprint map. Some works utilized only the sequence of magnetic field measurements with pattern recognition techniques [35,36,37,38,39]. In [24], the authors utilized the fingerprints of the changes in the raw magnetic field during indoor localization, but the performance can be largely affected by local distortions. Subbu et al. proposed an indoor localization solution by classifying signatures based on their patterns, using a dynamic time warping (DTW)-based approach [35]. The evaluation was performed using fingerprints containing simulated signatures of different ferromagnetic objects. In [36], an indoor positioning technique was proposed, which utilizes the differences in 2D magnetic field measurements that are collected over a distance with a pattern-matching algorithm. A 3D accelerometer was used to find the vertical direction. The authors assumed the following: the attitude variation of the smartphone is small; the change in the user velocity occurs only locally and sparsely; and the indoor environment is considered with no significantly varying magnetic field. In [37], a 2D geomagnetic fingerprint-based method was proposed, which uses point-to-point fingerprint matching. The method does not require an indoor map since it generates an indoor geomagnetic map, including all the turning points and connecting links with a crowdsourcing data collection module. The method also includes step and turning detection based on inertial sensors. The results based on experiments in a five-floor office building showed an average error smaller than 1.5 m. Ashraf et al. used geomagnetic field patterns with convolutional neural networks to perform indoor localization [38]. The performance was evaluated using measurements in two buildings with different experimental environments and path geometry. The results demonstrated that the users could be localized within 1.01 m at 75%. In [39], the magnetic field positioning performance of different machine learning methods was tested using pre-processed raw magnetic data. The test area was a corridor, where magnetic fingerprints were captured for 30 points.

The magnetic sensor-based technology was also used for the localization of mobile robots. In [25], global self-localization was considered for a mobile robot in one dimension using only a three-axis magnetometer. The Monte Carlo localization (MCL)-based technique was tested in experiments conducted in the corridors of four buildings. Both the three-dimensional measurement vector and the magnitude were tested in the method. Lee et al. proposed an indoor positioning system that recognizes magnetic sequence patterns by using a deep neural network [40]. The location was estimated by detecting the patterns of landmarks, using features extracted from the magnetic sequences. The achieved accuracy was 0.8 m in a corridor and 2.3 m in an atrium. In [41], magnetic fingerprints collected using a vision-based motion capture system were used together with odometry data in the localization framework. A particle filter-based method was proposed and the reported average errors achieved were 9 cm. The authors of [42] proposed a deep neural network-based method, which utilizes magnetometer measurement sequences, and applied it for both human and mobile robot localization. The method was tested with two public datasets and provided a localization error of around 1 m for both setups.

The disturbances in the magnetic field measured at different points can be used to form a fingerprint equivalent which is used in RSSI-based methods and carries additional information that can improve the efficiency of the RSSI-based fingerprinting. This study deals with indoor positioning using RSSI and magnetometer data together in a fingerprinting-based system, and the evaluation of the improvement provided by the proposed data fusion approaches. The contributions of this work can be summarized as follows:

- A novel 2D fingerprint-based positioning method is proposed, which fuses both the RSSI and magnetometer fingerprints using multilayer perceptron (MLP) neural networks. The method utilizes fingerprints measured in one plane near to the ground, since the goal is to provide absolute position information for the sensor fusion framework of a mobile robot. To the authors’ best knowledge, no such method was previously proposed.
- The proposed method is validated using measurements collected in two different indoor scenarios. Both scenarios are realistic since they include static obstacles.
- Three different combinations of magnetometer data are tested in the study. In the first version, the magnetometer measurements of the three sensor axes are tested. The second version utilizes the Z-axis measurements together with the magnetic field magnitudes in the X-Y plane, while the 3D magnitudes are utilized in the third version.
- The results obtained using the different fusion versions are compared with the results provided by utilizing only RSSI or magnetometer data to examine the improvement caused by the fusion of two data types.

The rest of the paper is organized as follows. Section 2 presents the proposed fingerprinting-based method. The applied measurement data are presented in Section 3. The experimental results are discussed in Section 4, while Section 5 summarizes the results of the paper.

## 2. Position Estimation Using the Fusion of RSSI and Magnetic Fingerprints

The aim of the proposed positioning method is to fuse RSSI and magnetometer data, thereby improving the overall positioning performance. 

### 2.1. RSSI-Based Positioning

RSSI is the calculation of real signal power received by a receiver and is typically expressed in decibel milliwatts (dBm) [10]. This measure can be used to measure the distance between transmitter and receiver devices, based on the transmitted and received signal power differences. RSSI measurements can be used in two ways in positioning methods. In geometric approaches, distances are calculated from the RSSI values using an adequate model. Two propagation models have been generally used to convert the RSSI to distance: the free-space models and the log-normal models. The free-space models are simple, but they do not consider the obstacles between receivers and transmitters, so they are often limited in real applications. The log-normal models are more suitable for different applications due to their flexibility. In fingerprinting-based techniques, the measured RSSIs of access points (AP) collected at given points of the area of interest are used to form the fingerprint database. This fingerprint database is used with a pattern recognition or pattern-matching algorithm, which can be later used to determine the position of a point given by a new measurement vector. Although geometric approaches are largely affected by obstacles, they do not have an effect on fingerprint-based techniques if they are static.

### 2.2. Magnetic Fingerprints

Vector magnetometers measure the magnetic field in three dimensions, and they are mainly used as compasses based on Earth’s magnetic field. The measurements of these sensors are affected by different factors, which can be classified into two groups. The first group includes deterministic errors, which occur due to manufacturing imperfections, and can be compensated for by calibrating the sensors. Such errors are the scale factor, the bias, and the non-orthogonality error. Magnetic sensors are also affected by external magnetic influences, which are caused by materials that generate or distort the magnetic field. These are called hard iron and soft iron effects [22,43,44]. Hard iron errors are time-invariant, undesired magnetic fields generated by ferromagnetic materials with permanent magnetism, which are additive to Earth’s magnetic field. The hard iron distortion is modeled by a 3×1 vector (***b****_HI_*). Soft iron distortion is the result of a material that influences or distorts a magnetic field, but does not necessarily generate a magnetic field itself, and is therefore not additive. The effect of the soft iron distortion is modeled by a 3×3 matrix (***A****_SI_*). The sensor model supposing that the deterministic errors are calibrated can be given by Equation (1).

(1) $$B=A_{SI}m+b_{HI},$$

where $B={\left[\begin{array}{ccc} B_{x} & B_{y} & B_{z} \end{array}\right]}^{T}$ is the measured output vector, while $m={\left[\begin{array}{ccc} m_{x} & m_{y} & m_{z} \end{array}\right]}^{T}$ is the magnetic field vector.

In an indoor environment, the measured magnetic field affected by the distortions at different points can be used to construct a fingerprint of an indoor space, which can be applied during localization.

### 2.3. Proposed Positioning Method

**Figure 1:**
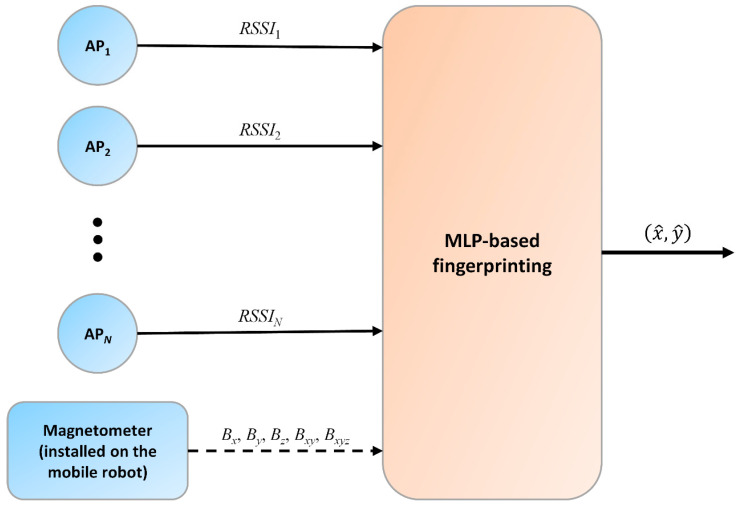
Architecture of the proposed method.

The magnetic sensor readings cannot be used for distance calculation, but the provided measurements of points with a known position can be utilized to construct a multidimensional fingerprint. Thus, it is obvious that the RSSI and magnetometer data should be fused in a fingerprinting-based method. The proposed method, which can be seen in Figure 1, utilizes together the fingerprints of *RSSI* values and the fingerprints of magnetometer measurements to train offline the MLP neural network. This trained MLP can be used in real-time to compute the $\left(\hat{x},\hat{y}\right)$ position of an unknown point based on the measurements collected at the given point. The used *RSSI* values are measured between APs with a known position and the mobile robot, while the magnetic field measurements are provided by a magnetometer installed on the mobile robot. In Figure 1, the magnetometer is connected with a dashed line to the fingerprinting algorithm since different versions of listed data are tested.

#### 2.3.1. Magnetometer Data

In total, three different possibly usable versions of magnetometer data are tested in the analysis:

- The first version utilizes the measurements on the three sensor axes, i.e., the *B_x_*, *B_y_*, and *B_z_* measurements. This kind of application of the magnetometer readings is only possible if the orientation of the sensor in the global coordinate frame is known for the given point, since different orientations at the same location result in different sensor readings. This version can be used to examine the most achievable positioning performance of the proposed method, since it contains the most information.
- In the second version, the 3D magnetic field magnitude (*B_xyz_*), which can be computed from the sensor outputs using Equation (2), is utilized since it is orientation independent.

(2) $$B_{xyz}=\sqrt{B_{x}^{2}+B_{y}^{2}+B_{z}^{2}},$$

- If the mobile robot is moving on a flat surface, then the degree of freedom decreases to three, i.e., (X, Y, θ). This makes the measurements in the Z-axis directly usable. The magnetic field magnitude in the X-Y plane (*B_xy_*), which can be calculated using Equation (3), can also be utilized without the knowledge of the θ angle. Thus, this version uses together the *B_z_* and the *B_xy_*.

(3) $$B_{xy}=\sqrt{B_{x}^{2}+B_{y}^{2}},$$

#### 2.3.2. Fingerprinting Algorithm

The proposed method applies MLP neural networks to perform positioning in 2D. The MLP is a widely used technique and it has been shown to provide the best results in RSSI-based fingerprint tasks compared to well-known methods, such as the weighted K-nearest neighbor (WKNN) and the random forest (RF) algorithms [45]. 

The method applies measurements collected at grid points of an indoor space to train the MLP. The measurement database of RSSI data used during the training process consists of WiFi $RSSI_{i,\left(x_{c},y_{c}\right)}$ measurements for i=1, 2, …, N APs. The measurements collected in a grid based on the $\left(x_{c},y_{c}\right)$ coordinates form a fingerprint for each AP. Similarly, the $B_{x,\left(x_{c},y_{c}\right)},B_{y,\left(x_{c},y_{c}\right)}$, and $B_{z,\left(x_{c},y_{c}\right)}$ magnetometer measurements taken at the grid points and the computed $B_{xy,\left(x_{c},y_{c}\right)}$, and $B_{xyz,\left(x_{c},y_{c}\right)}$ magnitudes also form fingerprints. These fingerprints for the different data types are also utilized during the MLP training. 

The developed MLP contains three layers: an input layer, a hidden layer, and an output layer. An input vector is formed for each given location from the corresponding $RSSI_{i}$ values measured between the mobile node and the *N* APs, together with the magnetic field strengths used in different versions. The output layer has two neurons, which provide the X and Y coordinates. For a given point in the training datasets, the target values corresponding to the input vector are defined by the $\left(x_{c},y_{c}\right)$ coordinates. The optimal number of neurons in the hidden layer should be defined by testing different configurations. The proposed MLP uses tangent sigmoid activation functions in the hidden layer and linear transfer functions in the output layer.

## 3. Applied Measurement Data

The proposed method was validated using measurements collected in two different scenarios. In the case of both scenarios, RSSI values were measured between multiple anchor nodes with a known position and one mobile node. The WiFi was chosen to provide the RSSI measurements, since it is the most suitable and popular wireless standard, and it is widely used in indoor localization methods [10,46]. The WiFi has high bitrate, high scalability, and is relatively less affected by external factors compared to other wireless standards, such as Bluetooth low energy (BLE), ZigBee, LoRaWAN, radio frequency identification (RFID), and ultra-wideband (UWB). Other technologies also have their own advantages and disadvantages. BLE is also a widely used technology in positioning applications [47,48]. The advantages of this technology are its low power consumption and fast connection establishment between the modules, but the lower range is a big disadvantage in the case of this technology [49]. Passive RFID technology-based systems are also popular in localization applications. The main advantage of these systems is their low cost, but their operation range is limited [50], which results in a high number of used tags [51]. The UWB technology provides high precision and low loss [52], but the higher cost is a big disadvantage of these systems.

Magnetometer measurements were also collected in the case of the mobile node. The measurements were taken in one plane near to the ground, which would be the case if the system was used with a mobile robot. Also, the disturbance sources are closer to the sensor, so they provide greater effects.

### 3.1. Measurement System

The applied measurement system consists of five anchor nodes (*N* = 5) and one mobile node. All six units are based on a NodeMCU ESP-32S V1.1 microcontroller board, which is capable of WiFi communication. The mobile node is also equipped with a three-axis magnetic sensor.

The used magnetometer is an HMC5883L type three-axis digital compass IC, which utilizes Honeywell’s anisotropic magnetoresistive (AMR) technology. The directional sensors feature precision in-axis sensitivity and linearity, and very low cross-axis sensitivity. The measurement range of the magnetic sensor is ±810 μT in 12-bit resolution with a 160 Hz maximal sampling rate. It also contains an I2C interface, which can be used to both configure the sensors and read the measurement values.

Since the data were processed offline, a central unit was used to collect the measurement data. The mobile node forwarded the magnetometer readings and the RSSI values read from the transceiver of the ESP32 board during communication with the APs.

### 3.2. Data Acquisition

In the case of both scenarios, the measurements were collected at defined grid points. At all points, 10 RSSI measurements were recorded for all APs and 10 magnetometer measurements were also collected. The 10 samples were later averaged to decrease the effect of noise.

During the data acquisition process, the WiFi channel was set to 1 and the antenna Tx power was 19.5 dBm in the case of all wireless modules.

The version where the measurements of the three magnetometer axes are utilized required the knowledge of the mobile unit’s orientation. To obtain usable magnetometer data for this version, the orientation of the mobile unit was set to as constant as possible at different points.

Besides the hard and soft iron effects, the magnetometer measurements are also affected by deterministic errors, such as scale factors, bias, and non-orthogonality errors. To compensate for these effects, the magnetometer was calibrated using an evolutionary algorithm-based calibration method, which utilizes measurements taken in multiple orientations to compute the calibration parameters [53]. This process was performed before data acquisition.

#### 3.2.1. First Scenario

**Figure 2:**
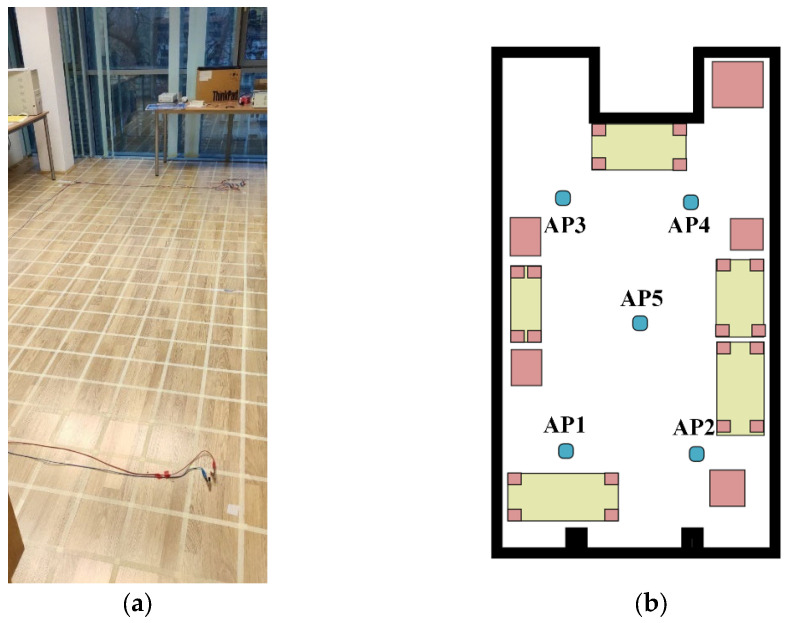
Measurement setup of the first scenario: (**a**) environment; (**b**) schematic drawing.

In the first scenario, a smaller room was chosen, which had enclosing dimensions of 6.6 m × 3.6 m. Figure 2 shows the measurement environment in the room and its schematic drawing with the position of the anchor nodes. To make the setup realistic, multiple static objects were left in the room, such as cabinets and tables. It is also important to note that one of the walls was made from glass, moreover a concrete column could also be found in the room. Some measurement points were none-line-of-sight (NLOS) due to the objects.

**Figure 3:**
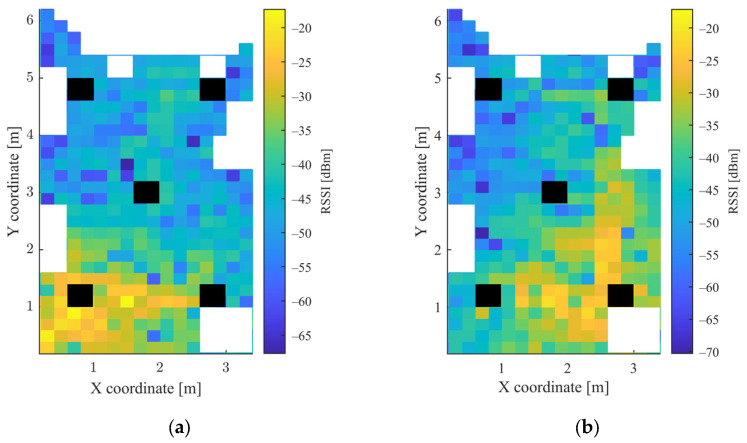

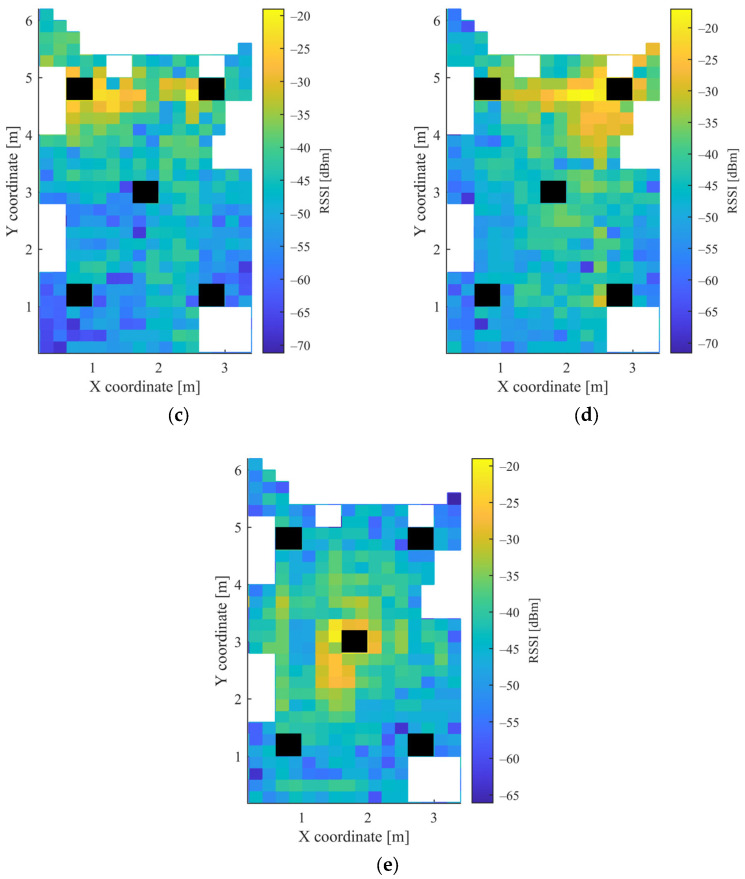
Heatmap of RSSI values in the first scenario for: (**a**) AP1; (**b**) AP2; (**c**) AP3; (**d**) AP4; and (**e**) AP5.

**Figure 4:**
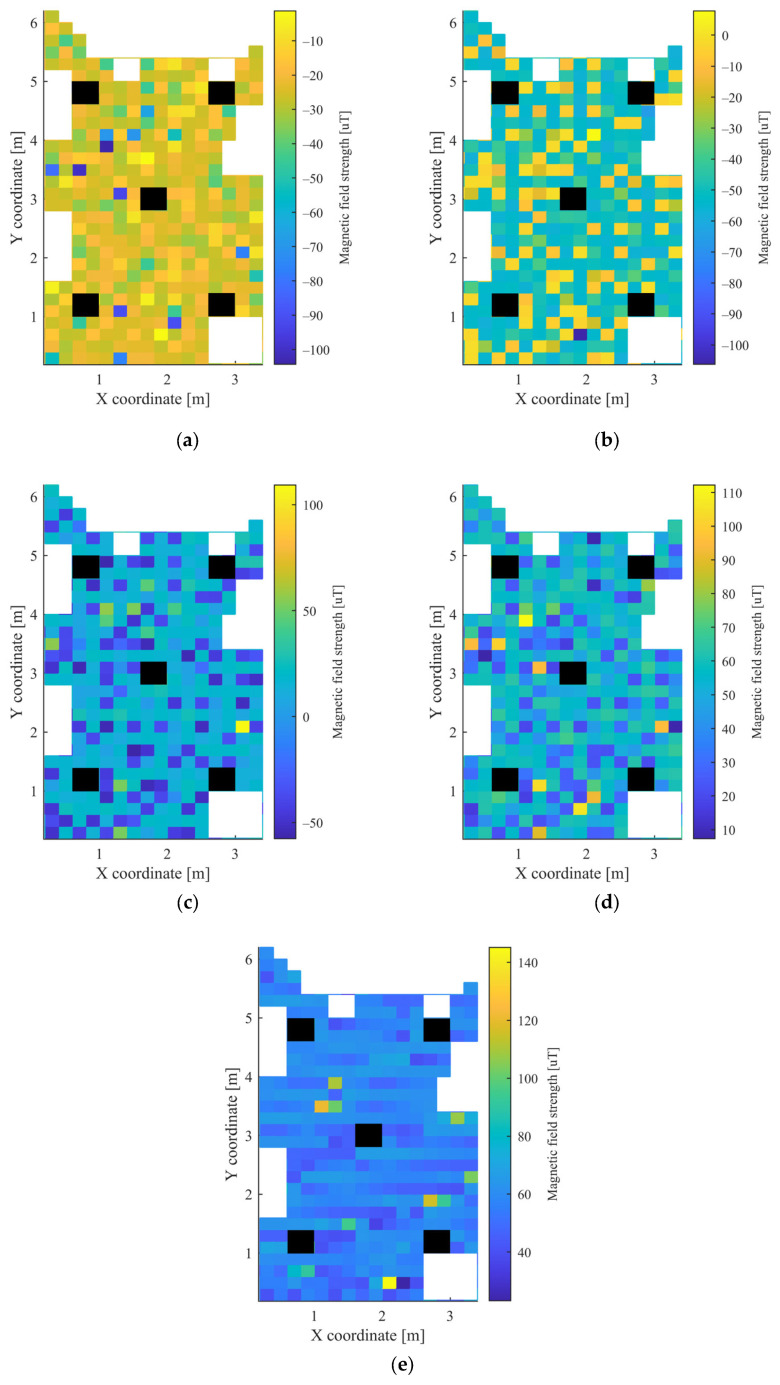
Heatmap of the magnetic field strength in the first scenario for: (**a**) *B_x_*; (**b**) *B_y_*; (**c**) *B_z_*; (**d**) *B_xy_*; and (**e**) *B_xyz_*.

In the grid, which was used to determine the measurement points, squares with a side length of 20 cm were defined. The height of both the mobile unit and the APs was 4 cm. Altogether 426 points were used for data acquisition. The mobile node was moved manually between the measurement points and there was no one in the room during the measurements. The measurements were collected over the course of one day. The heatmaps of the collected RSSI measurements for the 5 APs and the magnetometer data are shown in Figure 3 and Figure 4, respectively. The white parts in the figures represent the places where measurements could not be taken due to the obstacles, while the black rectangles show the positions of the APs.

#### 3.2.2. Second Scenario

**Figure 5:**
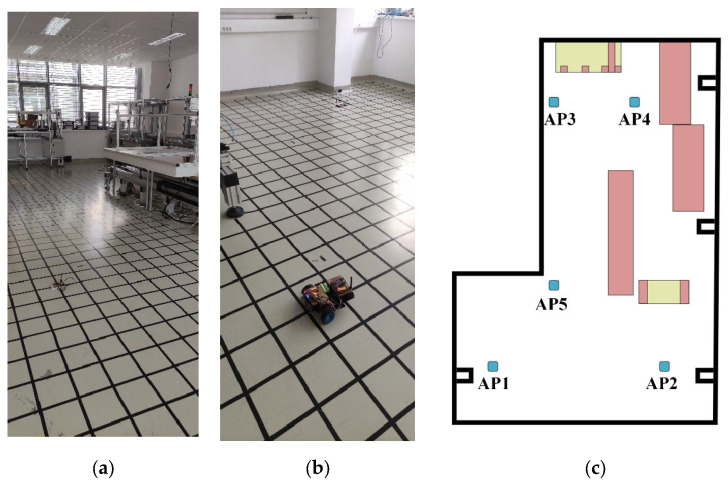
Measurement setup of the second scenario: (**a**) environment of the laboratory; (**b**) environment with the mobile robot; and (**c**) schematic drawing.

The measurements of the second scenario were collected in a larger laboratory with enclosing dimensions of 12 m × 8 m. Both the measurement environment in the laboratory and its schematic drawing with the position of the APs can be seen in Figure 5. Similarly to the first scenario, static objects were present in the laboratory during the measurements, such as tables, robotic cells, and a linear rail with a robotic arm. Due to the objects, many points were NLOS to some APs.

**Figure 6:**
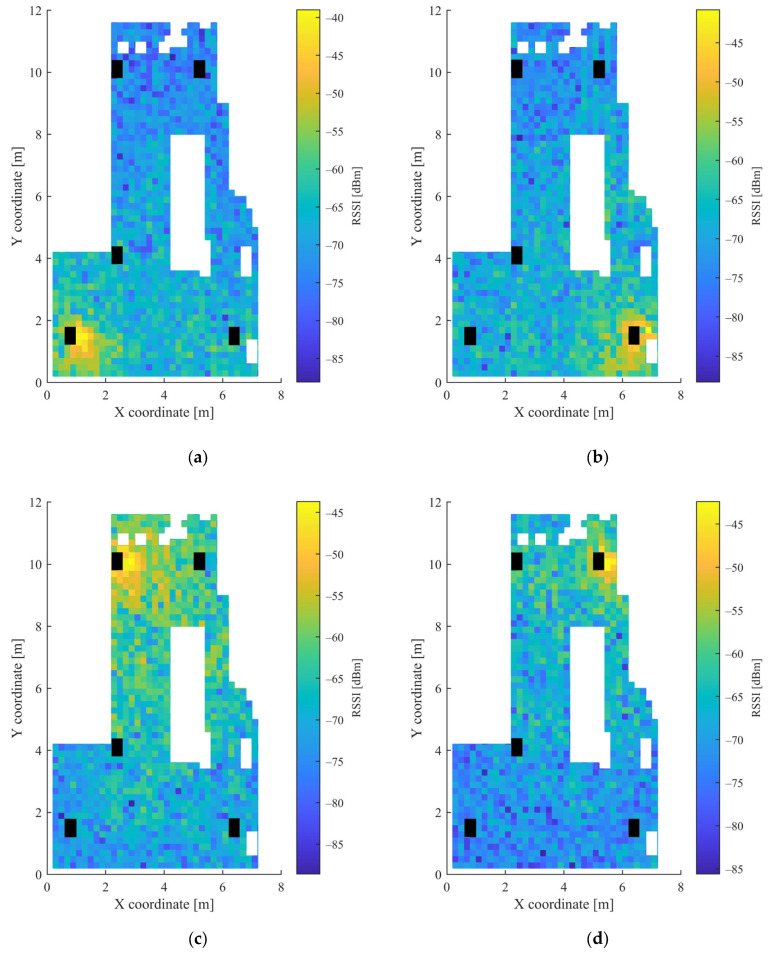

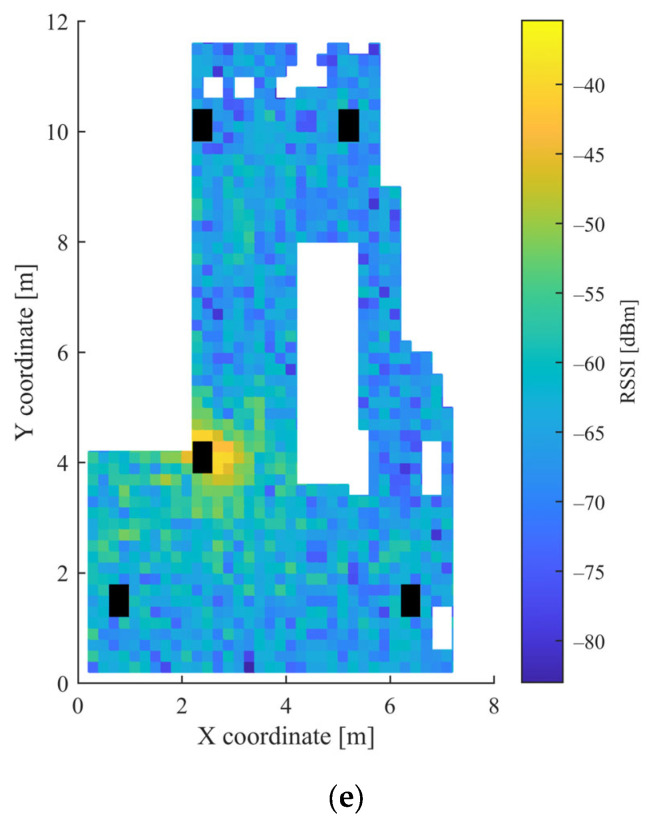
Heatmap of RSSI values in the second scenario for: (**a**) AP1; (**b**) AP2; (**c**) AP3; (**d**) AP4; and (**e**) AP5.

**Figure 7:**
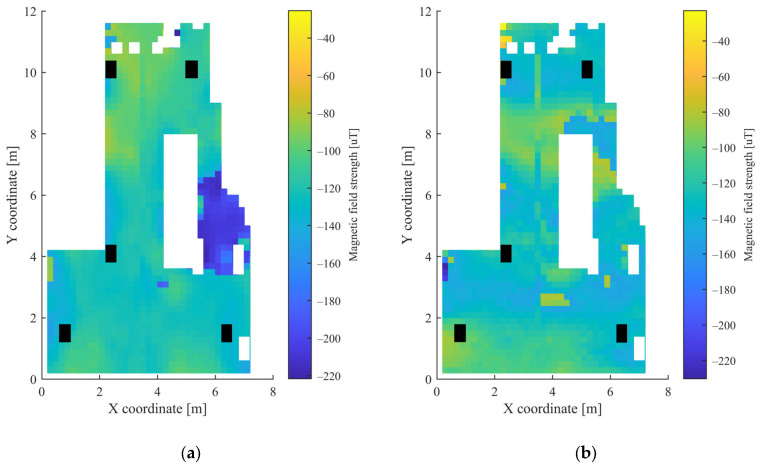

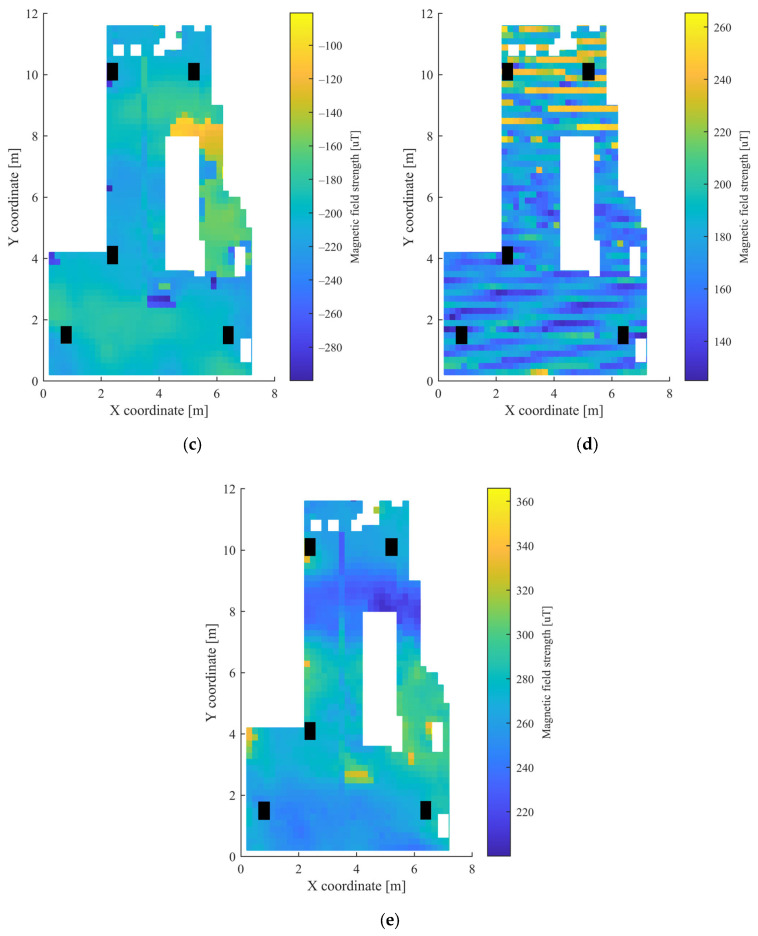
Heatmap of the magnetic field strength in the second scenario for: (**a**) *B_x_*; (**b**) *B_y_*; (**c**) *B_z_*; (**d**) *B_xy_*; and (**e**) *B_xyz_*.

The grid was also defined using 20 cm distances, as in the first scenario. Due to the high number of points, the movement of the measurement unit between the grid points was realized using a mobile robot. The height of the mobile unit and the APs was 10.8 cm and 3.5 cm, respectively. Altogether, measurements were collected at 1408 points, defined by the grid. Further measurements were taken at 20 random positions, which were later used for the testing of the trained MLPs. These points were defined by random coordinates and were not identical with any grid point coordinates. The measurements were collected over the course of one day. Figure 6 shows the heatmaps of the RSSI values from the 5 APs, while the heatmaps of the magnetic sensor data can be seen in Figure 7. The white parts in both Figure 6 and Figure 7 represent the places where measurements could not be taken due to the obstacles, while black parts show the places of the APs.

## 4. Experimental Results

**Figure 8:**
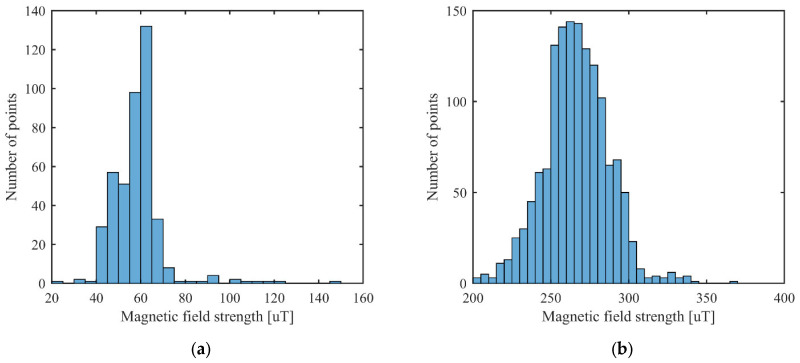
Histogram of the *B_xyz_* magnetic field magnitude for: (**a**) first scenario; and (**b**) second scenario.

It can be observed from Figure 3 and Figure 4 for the first scenario and from Figure 6 and Figure 7 for the second scenario, that the heatmaps of the RSSI data and magnetic field strengths carry additional information when compared to each other. The histogram of the *B_xyz_* magnetic field magnitude for the two scenarios can be seen in Figure 8. The histograms show that the variance of the magnetometer measurements is much higher in the case of the second scenario, which is the laboratory.

### 4.1. Datasets and MLP Training

The data measured at the grid points were utilized to train the MLP neural networks separately for the two scenarios. Since no test points were determined in the first scenario, two separate datasets were defined. In the first dataset, all points were utilized as training data without test data to examine the obtainable error levels at the grid points. In the second dataset, a subset of points containing every second point was used in the training process, while the remaining 50% of the points were applied for the testing of the trained MLPs.

Altogether, seven combinations of used data were tested to examine their performance in the two scenarios. The tested combinations are listed as follows:

1. *RSSI*
2. *B_x_*, *B_y_*, *B_z_*
3. *B_xy_*, *B_z_*
4. *B_xyz_*
5. *RSSI*, *B_x_*, *B_y_*, *B_z_*
6. *RSSI*, *B_xy_*, *B_z_*
7. *RSSI*, *B_xyz_*

The training of the MLP neural networks for all datasets was tested with 1–100 hidden layer neurons to find the necessary configuration. Since the achievable error rates largely depend on the initial weights, all configurations were tested 10 times and the results containing the lowest error rates were used later in the evaluation process. Multiple performance metrics were utilized in the evaluation process. The mean absolute error (MAE) was used as the main metric, which can be calculated using Equation (4),

(4) $$\mathrm{MAE}=\frac{1}{M}\sum_{i=1}^{M}E_{i},$$

where *M* is the number of points, while E*_i_* is the error corresponding to the *i*th point, which can be calculated using Equation (5).

(5) $$E_{i}=\sqrt{{\left(\hat{x}-x_{c}\right)}^{2}+{\left(\hat{y}-y_{c}\right)}^{2}}$$

In Equation (5), $\hat{x}$ and $\hat{y}$ are the determined coordinates, while $x_{c}$ and $y_{c}$ are the real position of the *i*th point.

Other metrics corresponding to the lowest achieved MAE were also computed. These were the standard deviation (STD) and the root mean squared error (RMSE), which can be calculated using Equations (6) and (7), respectively.

(6) $$\mathrm{STD}=\sqrt[{}]{\frac{1}{M-1}\overset{M}{\underset{i=1}{\sum }}{\left(E_{i}-\mathrm{MAE}\right)}^{2}}$$

(7) $$\mathrm{RMSE}=\sqrt{\frac{\sum_{i=1}^{M}E_{i}^{2}}{M}}\;$$

The hyperparameters of the MLP training process can be found in Table 1. The training was done offline in MATLAB on a PC with the following specifications: Intel Core i5-12600K 3.69 GHz CPU, 16 GB DDR4 3600 MHz RAM, M2 SSD, ASUS GeForce RTX 3080 10GB GPU. The training process was time consuming since multiple hours were required, even for one version. Although it is a complex and time-consuming process to find the optimal network for a given version, it does not affect the real-time operation after implementation.

### 4.2. Evaluation of the First Scenario

The best achieved MAE results and the corresponding STD and RMSE results, using different versions of data for the two datasets in the case of the first scenario, are summarized in Table 2. It can be observed from the results that using the proposed method, the errors significantly decrease in the case of the training data. Using datasets where all points were used as training data, significant improvements were achieved using the versions that utilized *RSSI*, *B_x_*, *B_y_*, *B_z_* and *RSSI*, *B_xy_*, *B_z_*, compared to results where only *RSSI* was used. Here, the MAE decreased from 50.71 cm to 34.64 cm and 41.68 cm, respectively. In the datasets where every second point was utilized, the MAE using only *RSSI* was more than 10 cm lower than when all points were used. The improvement caused by the fusion with magnetometer data was 7–10 cm, using any version. It should be noticed that when using the *RSSI*, *B_xy_*, *B_z_* version, an MAE more than 2 cm lower was achieved than with the other two fusion-based versions. In the case of test data, no improvement can be noticed since the *RSSI* and the fusion-based versions provided nearly the same results. This can be caused by the low variance of the magnetic field measurements in this scenario, and the large distances between the points due to the usage of every second point. The achieved results using only magnetometer data in this scenario show a much lower performance than the other versions.

**Figure 9:**
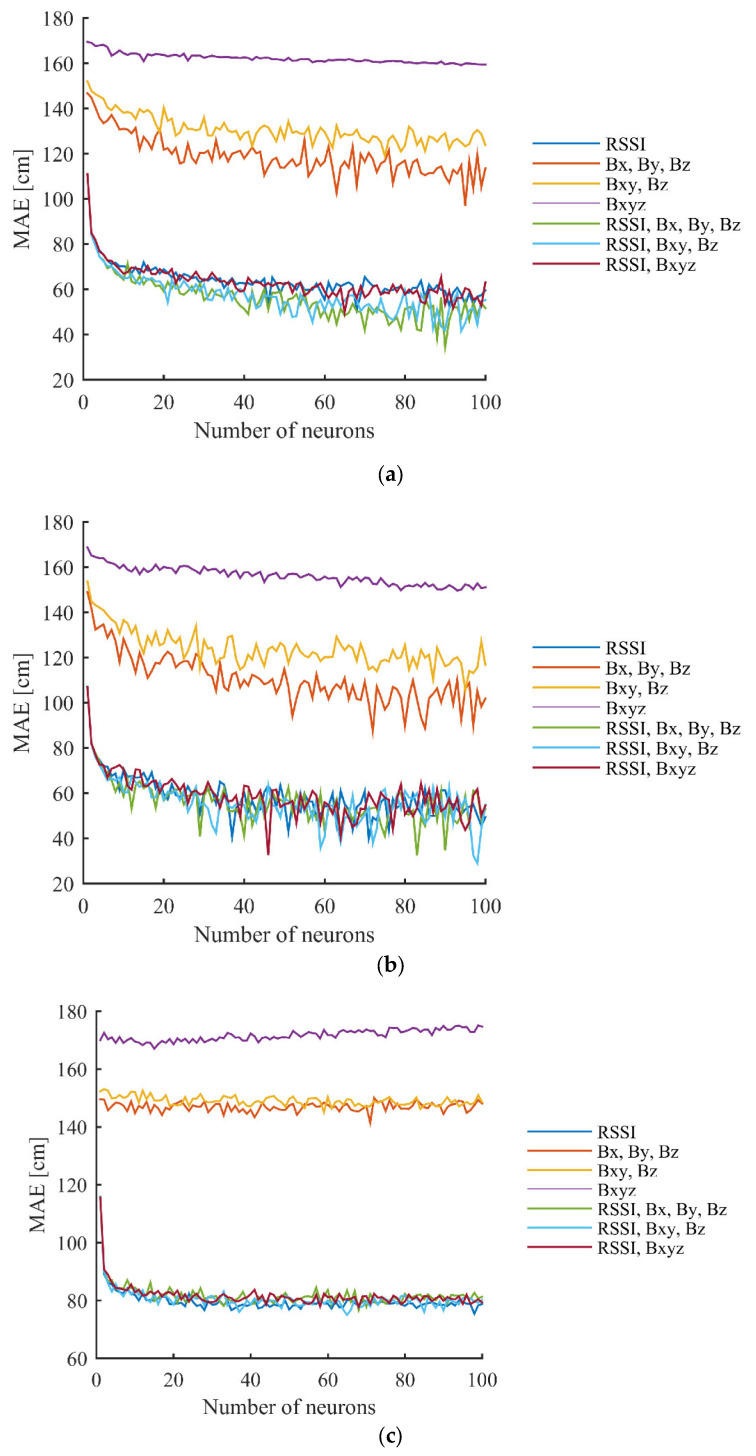
Results achieved in the first scenario using different number of hidden layer neurons with different versions of used data for: (**a**) all points in the grid (training data); (**b**) every second point in the grid (training data); and (**c**) every second point in the grid (test data).

Figure 9 shows the best achieved MAE using different setups, based on the hidden layer neuron numbers with different versions of used data. It can be seen from the results that the MAE starts to converge around 40–50 neurons in the case of the training data. Only slight improvements can be noticed with higher neuron numbers. In the case of the test data, the MAE converges around 20 neurons.

**Figure 10:**
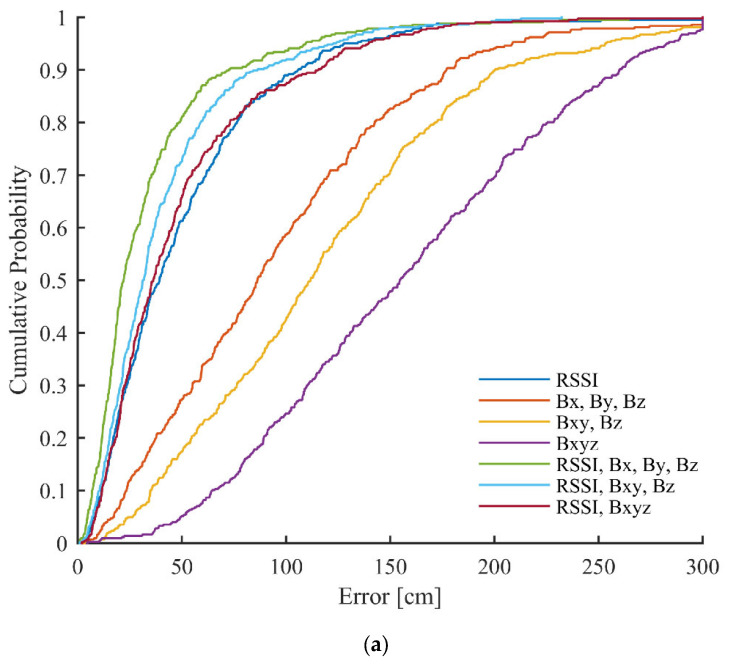

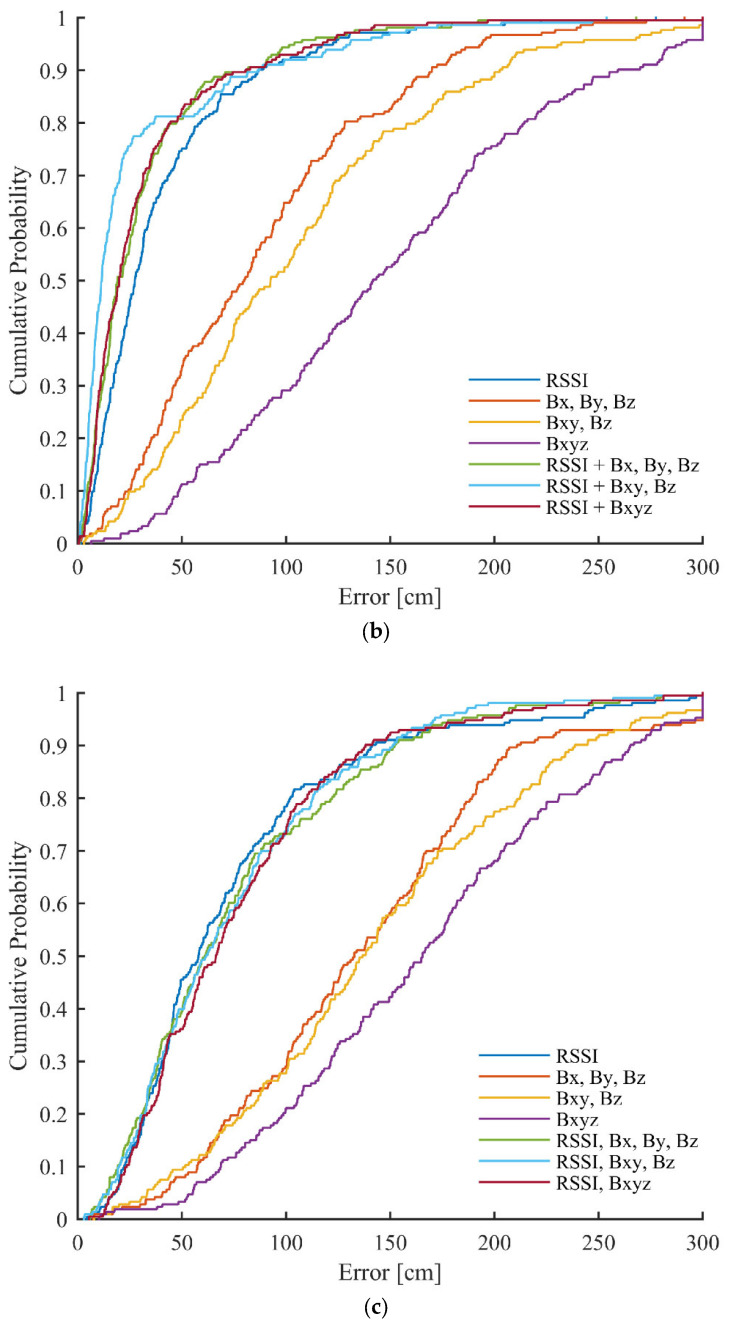
CDFs of errors achieved in the first scenario with different versions of used data for: (**a**) all points in the grid (training data); (**b**) every second point in the grid (training data); and (**c**) every second point in the grid (test data).

The cumulative density functions (CDF) of errors for the different used versions of data can be seen in Figure 10. The errors larger than 300 cm were truncated. It can be noticed that the proposed method significantly improves the error distribution in the case of the training data. For example, when using all grid points for training, around 60% of points give below 50 cm errors with only *RSSI*, which increases to ~70% and ~80% using *RSSI*, *B_xy_*, *B_z_* and *RSSI*, *B_x_*, *B_y_*, *B_z_*, respectively. With the test data, the fused versions provide almost identical curves, as with using only *RSSI*.

### 4.3. Evaluation of the Second Scenario

Table 3 presents the best achieved MAE and the corresponding STD and RMSE results in the second scenario for the different combinations of used data. Much higher improvements can be noticed with the proposed method compared to the results achieved in the first scenario, which is obviously the cause of the higher variance of the magnetic field readings. It is very important to note that approximately 20 cm better results were obtained in the case of both the training and test data using *B_x_*, *B_y_*, *B_z_*, compared to the results achieved using only *RSSI*. It is also important to notice that the MAE for almost all versions was lower in the case of the test data than with the training data. This can be caused by lower error rates in the areas where the random locations were defined. The proposed method resulted in significantly lower error rates for all three versions compared to both *RSSI* and *B_x_*, *B_y_*, *B_z_*, in the case of both the training and test data. The best results were provided by the version utilizing *RSSI*, *B_x_*, *B_y_*, *B_z_*, for which the achieved MAE was 99.59 cm and 77.27 cm for the training and test data, respectively. The improvement with this combination of used data resulted in more than 35% improvement compared to the results obtained using only *RSSI* or *B_x_*, *B_y_*, *B_z_*. The version using *RSSI*, *B_xy_*, *B_z_*, which can be used in the case of flat surfaces without knowledge of orientation, resulted in approximately 10 cm worse results than using *RSSI* and the measurements of the three sensor axes together. Compared to the results based on only *RSSI*, this version resulted in more than 35 % improvement. The worst results of the three fused versions were provided by utilizing *RSSI* with the 3D magnetic field magnitude together. The provided results with this orientation-independent version were 128.96 cm for the training data and 126.25 cm for the test data. Compared to the *RSSI*-based results, the improvement was 25.37% and 16.71%, respectively. 

**Figure 11:**
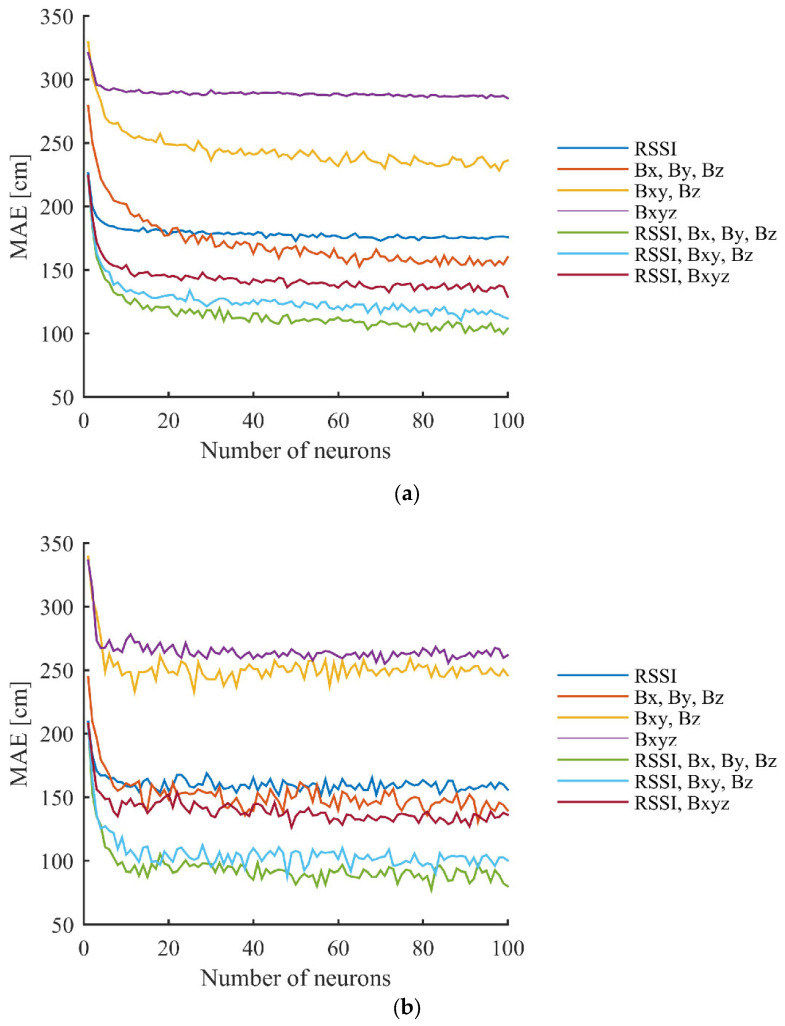
Achieved results in the second scenario using different number of hidden layer neurons with different versions of used data for: (**a**) grid points (training data); and (**b**) test points.

The achieved MAE using a different number of neurons in the hidden layer, for the various versions of used data recorded in the second scenario, can be seen in Figure 11. It can be seen from the results that most versions converge with 30–40 neurons in the case of the training data and with 20 neurons using the test data, but slight improvements can also be noticed with a higher number of hidden layer neurons in both cases.

**Figure 12:**
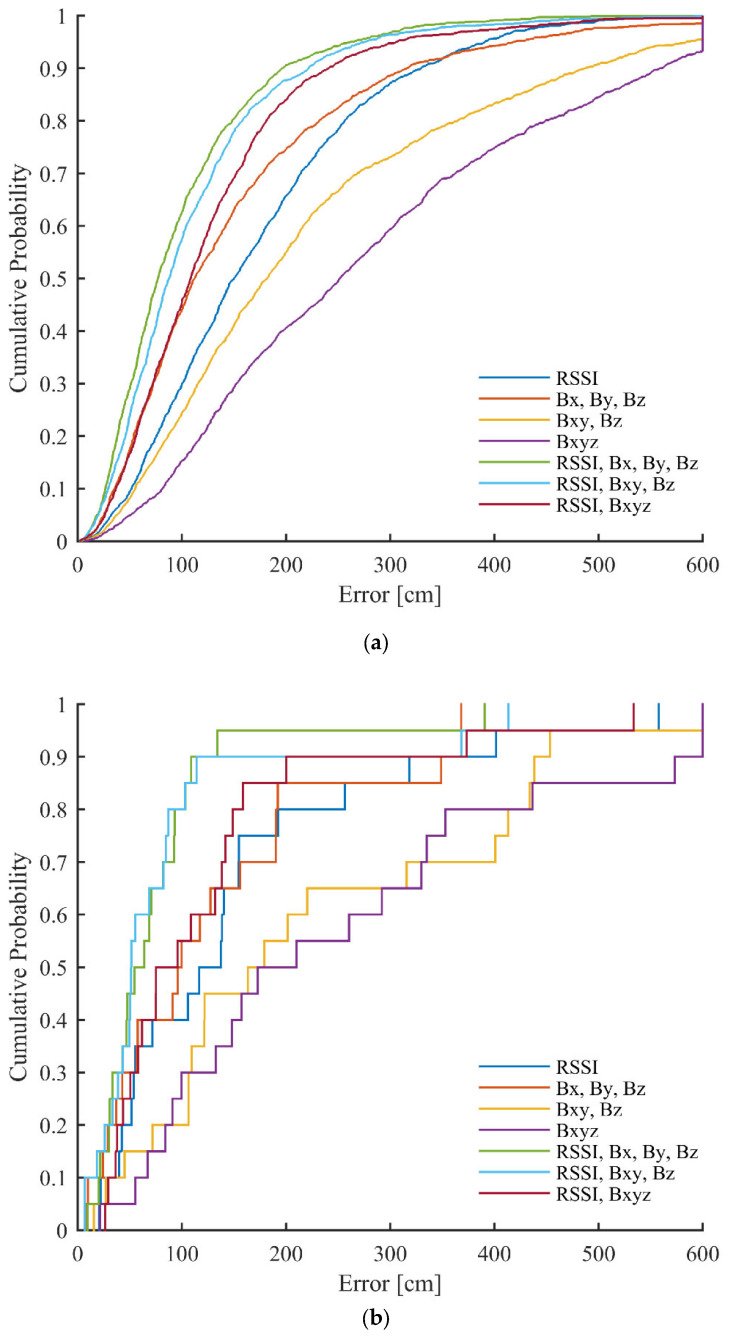
CDFs of errors achieved in the first scenario with different versions of used data for: (**a**) grid points (training data); and (**b**) test points.

The CDFs of error for the best setups, using different versions of used data in the case of the second scenario, can be seen in Figure 12. The errors larger than 600 cm were truncated. It can be observed from the results that significant improvements can be achieved in the error distributions using the different versions of the proposed method.

## 5. Conclusions

In this work, a novel fingerprinting-based positioning method was proposed, which fuses the RSSI data measured between anchor nodes and a mobile node with the magnetic field measurements collected from the mobile unit. In total, three different versions of used magnetometer data were tested in the proposed method. The position is determined using three-layer MLP neural networks.

The method was validated using data collected in two different realistic indoor scenarios, i.e., in a smaller room and a larger laboratory. Multiple static objects were present in both scenarios. The MLP ANNs were tested with a various number of hidden layer neurons to find the optimal configuration.

The achieved results show that significant improvement, even above 35%, can be achieved using the proposed method, compared to the results achieved using only the RSSI or magnetic sensor data, if the variance of the magnetic field measurements is high in the observed indoor space. In the first scenario, where the variance of magnetometer readings was lower, improvement could be noticed only in the case of the training data. In the second scenario, where the variance in the magnetic field strength was much higher, significant improvements were achieved. The best results were obtained by fusing RSSI data with magnetometer measurements in the three axes, but this version requires knowledge of the orientation of the mobile unit in the global coordinate frame. The version using the RSSI data, together with the magnetometer measurements in the Z-axis and the magnitude in the X-Y plane, provided more than 35% better results compared to only RSSI-based error rates. This version can be used in the case of flat surfaces without knowledge of the orientation. Utilizing the RSSI data together with the 3D magnetometer magnitudes resulted in approximately 25% and 15% improvement in the case of the training and test data, respectively. 

The limitations of the proposed approach include that the magnetic sensor needs to be close to the objects causing the disturbances. Another limitation is that some versions depend on the orientation, which should be solved by implementing an orientation estimation framework that can provide the necessary information. Further future goals include achieving further improvements in the error rates by incorporating other data and its application in a sensor fusion-based localization framework.

## Figures and Tables

**Table 1:** Hyperparameters of the MLP training process.

Hyperparameter	Value
training function	Levenberg–Marquardt backpropagation
performance function	mean squared error (MSE)
normalization	minmax to range [−1, +1]
ratio of data used for training	70%
ratio of data used for validation	30%
maximum number of epochs to train	6000
performance goal	0
maximum validation failures	20
minimum performance gradient	10^−7^
maximum time to train in seconds	inf

**Table 2:** Best achieved MAE and the corresponding STD and RMSE results for different datasets in the first scenario using different versions of data.

Used Data	All Points in the Grid (Training Data)		Every Second Point in the Grid (Training Data)		Every Second Point in the Grid (Test Data)	
	MAE ± STD [cm]	RMSE [cm]	MAE ± STD [cm]	RMSE [cm]	MAE ± STD [cm]	RMSE [cm]
*RSSI*	50.71 ± 44.05	67.13	40.04 ± 37.39	56.91	75.43 ± 58.12	97.06
*B_x_*, *B_y_*, *B_z_*	96.88 ± 60.38	118.01	87.48 ± 54.95	104.86	141.48 ± 76.47	161.10
*B_xy_*, *B_z_*	119.01 ± 65.28	138.07	105.52 ± 65.93	126.61	145.12 ± 73.04	163.54
*B_xyz_*	159.19 ± 74.25	175.72	149.67 ± 75.32	167.64	167.02 ± 76.12	183.48
*RSSI*, *B_x_*, *B_y_*, *B_z_*	34.64 ± 32.87	51.86	32.44 ± 37.27	49.35	76.67 ± 56.20	96.81
*RSSI*, *B_xy_*, *B_z_*	41.68 ± 36.58	55.42	29.07 ± 41.18	52.58	75.03 ± 55.33	93.14
*RSSI*, *B_xyz_*	49.86 ± 45.38	67.39	32.54 ± 39.17	50.85	77.79 ± 58.17	97.05

**Table 3:** Best achieved MAE and corresponding STD and RMSE results for different versions of used data in the second scenario.

Used Data	Grid Points (Training Data)		Test Points (Test Data)	
	MAE ± STD [cm]	RMSE [cm]	MAE ± STD [cm]	RMSE [cm]
*RSSI*	172.80 ± 111.14	205.43	151.58 ± 116.25	199.00
*B_x_*, *B_y_*, *B_z_*	152.67 ± 129.81	200.37	130.49 ± 113.16	172.51
*B_xy_*, *B_z_*	228.28 ± 166.67	284.65	232.86 ± 172.64	295.42
*B_xyz_*	285.20 ± 176.22	338.88	255.07 ± 193.52	317.23
*RSSI*, *B_x_*, *B_y_*, *B_z_*	99.59 ± 78.93	127.06	77.27 ± 76.75	110.47
*RSSI*, *B_xy_*, *B_z_*	110.19 ± 86.44	140.03	87.63 ± 94.27	137.01
*RSSI*, *B_xyz_*	128.96 ± 96.54	161.07	126.25 ± 115.66	175.58

## Data Availability

The data presented in this study are openly available at: https://github.com/petersarcevic/rssi_magnetometer_fingerprint_database (accessed on 30 January 2023).
